# Pulmonary responses in current smokers and ex-smokers following a two hour exposure at rest to clean air and fine ambient air particles

**DOI:** 10.1186/1743-8977-10-58

**Published:** 2013-11-18

**Authors:** Milan J Hazucha, Philip A Bromberg, John C Lay, William Bennett, Kirby Zeman, Neil E Alexis, Howard Kehrl, Ana G Rappold, Wayne E Cascio, Robert B Devlin

**Affiliations:** 1 Center for Environmental Medicine, Asthma and Lung Biology, University of North Carolina, CB#7310, 104 Mason Farm Road, Chapel Hill, NC 27599-7310, USA; 2 Department of Medicine, School of Medicine, University of North Carolina, CB#7020, Chapel Hill, NC 27599-7020, USA; 3 Department of Pediatrics, School of Medicine, University of North Carolina, Chapel Hill, NC 7220, USA; 4 Environmental Public Health Division, MD 58B, National Health and Environmental Effects Research Laboratory, U.S. EPA, Research Triangle Park, NC 27711, USA

**Keywords:** CAFP, Chamber exposure, Spirometry, Older smokers, Ex-smokers, DTPA clearance half-time, Lung diffusing capacity, Blood chemistry

## Abstract

**Background:**

Increased susceptibility of smokers to ambient PM may potentially promote development of COPD and accelerate already present disease.

**Objectives:**

To characterize the acute and subacute lung function response and inflammatory effects of controlled chamber exposure to concentrated ambient fine particles (CAFP) with MMAD ≤ 2.5 microns in ex-smokers and lifetime smokers.

**Methods:**

Eleven subjects, aged 35–74 years, came to the laboratory 5 times; a training day and two exposure days separated by at least 3 weeks, each with a post-exposure visit 22 h later. Double-blind and counterbalanced exposures to “clean air” (mean 1.5 ± 0.6 μg/m^3^) or CAFP (mean 108.7 ± 24.8 μg/m^3^ ) lasted 2 h with subjects at rest.

**Results:**

At 3 h post-exposure subjects’ DTPA clearance half-time significantly increased by 6.3 min per 100 μg/m^3^ of CAFP relative to “clean air”. At 22 h post-exposure they showed significant reduction of 4.3% per 100 μg/m^3^ in FEV_1_ and a significant D_L_CO decrease by 11.1% per 100 μg/m^3^ of CAFP relative to “clean air”. At both 3 h and 22 h the HDL cholesterol level significantly decreased by 4.5% and 4.1%, respectively. Other blood chemistries and markers of lung injury, inflammation and procoagulant activity were within the normal range of values at any condition.

**Conclusions:**

The results suggest that an acute 2 h resting exposure of smokers and ex-smokers to fine ambient particulate matter may transiently affect pulmonary function (spirometry and D_L_CO) and increase DTPA clearance half-time. Except for a post exposure decrease in HDL no other markers of pulmonary inflammation, prothrombotic activity and lung injury were significantly affected under the conditions of exposure.

## Background

Numerous field and epidemiological studies have shown associations between ambient particulate air pollution exposure and longitudinal changes in peak expiratory flow rates, respiratory symptoms, medication use, mortality and morbidity, including hospital admissions for cardiopulmonary disease [1]. Cigarette smoking can impair lung function and, therefore, smokers and ex-smokers may be more vulnerable to PM exposure [2]. Despite the concentrated effort by the American Lung Association**,** the American Heart Association and other agencies to reduce smoking by the US population recent trend estimates show that 1 in 5 adults still smoke. The prevalence rate of current smokers in some states is as high as 28% [3].

Because smoking is a leading cause of COPD, increased susceptibility of smokers to ambient PM may potentially promote development of COPD. The risk estimate for residents of high pollution areas of developing COPD was higher in past smokers than never smokers [4]. Middle-aged current smokers exposed to ambient PM showed a small but statistically significant negative association between pulmonary function (FEV_1_) and PM_10_ levels [5]. To date, very little is known about whether smokers are more susceptible to PM since only a few epidemiologic studies and no laboratory studies have explored the effects of ambient PM on smokers. Laboratory inhalation study of lung deposition of fine particles has shown an increased deposition in smokers which may potentially result in greater susceptibility to injury by ambient PM [6]. Controlled exposure studies and acute panel studies of nonsmokers have, except for a few reporting a post-exposure decrease in D_L_CO [7], generally failed to find any consistent lung function changes associated with exposure to PM [8,9].

The general objective of the study was to examine and characterize the acute and subacute lung function response and inflammatory effects of controlled exposure of middle-aged and older ex-smokers and lifetime smokers to concentrated ambient fine particles (CAFP). More specific objectives were to determine whether smokers following controlled exposure to CAFP develop (a) decrements in spirometry or D_L_CO, (b) increased respiratory epithelial permeability as measured by ^99m^Tc-DTPA clearance, and (c) changes in pro-coagulant factors and markers of inflammation, oxidative stress and lung injury in peripheral venous blood.

## Results

### Subjects

Table 1 shows the physical characteristics, smoking history expressed as packs per day per year (pack-years) and baseline pulmonary function of the study participants. All but two subjects (#1 and 2) who quit smoking 7 and 10 years ago, respectively, were current smokers.

**Table 1:** Physical characteristics and baseline pulmonary function

**Subj**	**Gender**	**Race**	**Age**	**Height**	**Weight**	**Smoking**	**BMI**	**BSA**	**FEV**_**1**_	**FEV**_**1**_	**FEV**_**1**_**/FVC**	**D**_**L**_CO**
**#**			**[yrs]**	**[cm]**	**[kg]**	**[pk-yr]**		**[m**^**2**^**]**	**L/s**	**[% pred]**	**[%]**	
1	M*	C	74	172	65.3	9.0	22.1	1.77	2.2	80.2	52.8	9.7
2	F*	C	63	157	60.0	40.0	24.3	1.60	1.3	61.3	58.5	16.3
3	F	C	39	157	114.6	48.0	46.5	2.10	1.6	60.9	57.0	20.3
4	M	C	50	172	77.3	31.0	26.1	1.90	3.5	103.2	78.4	25.3
5	M	B	42	183	75.8	39.0	22.6	1.97	3.3	92.9	72.0	35.5
6	F	B	40	159	87.1	38.0	34.4	1.89	1.9	84.1	82.9	30.0
7	F	C	46	163	61.3	30.0	23.1	1.66	2.9	109.8	81.7	17.1
8	F	B	55	160	60.6	54.0	23.7	1.63	2.3	114.1	75.0	13.7
9	F	C	35	168	82.1	39.0	29.1	1.92	3.7	123.0	71.5	22.6
10	F	B	53	154	55.8	60.0	23.5	1.53	1.6	83.6	82.2	8.7
11	F	B	35	165	82.7	12.0	30.4	1.90	2.4	96.5	78.2	15.1
Mean			48	165	74.8	27.8	36.4	1.80	2.5	91.8	72.3	20.0
SEM			4	3	5.2	2.2	4.7	0.10	0.2	6.1	3.5	2.6

*ex-smoker; **D_L_CO in ml/min/mm Hg;

M = male, F = female; C = Caucasian, B = black; BSA = body surface area; BMI = body mass index.

### Particle concentration

On one of the days each subject was exposed to clean filtered air (CA) with particle concentration ranging from 0.0–5.8 μg/m^3^ (mean 1.5 ± 0.6). Any gaseous pollutants were diluted by a factor of four. On the other day each subject was exposed to CAFP concentration ranging from 28.6 – 305.9 μg/m^3^ (mean 108.7 ± 24.8). Most of the particles came from traffic in the vicinity of the facility.

### Pulmonary function

**Figure 1:**
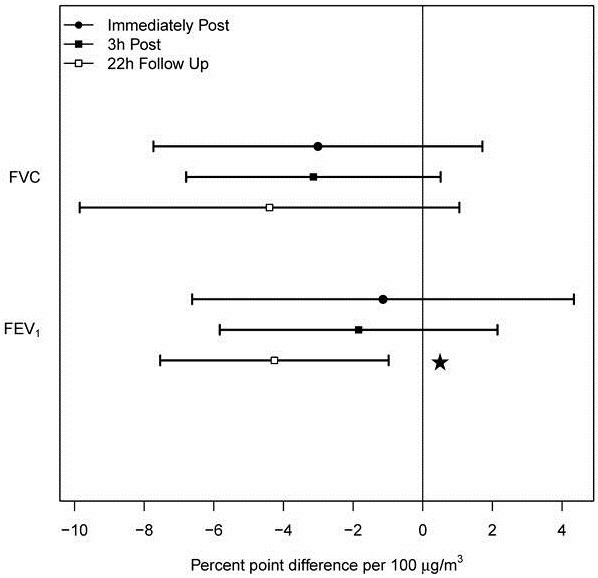
**Estimated differences between CAFP and CA exposures for spirometric endpoints (FVC and FEV**_**1**_**) measured immediately after and, at 3 h and 22 h after a two-hour exposure.** The estimates are expressed as % point differences per 100 μg/m^3^ increase in PM concentration relative to the pre-exposure level. Horizontal bars correspond to 95% confidence interval around the mean value. The asterisk indicates significant difference between CA and CAFP exposure (p = 0.008).

Immediately following exposure estimated difference in FVC and FEV_1_ response (Figure 1) between CA and CAFP was −3.0 and −1.1% points per 100 μg/m^3^ of CAFP, respectively. Three hours post-exposure this difference was −3.1 and −1.8% points, respectively_._ None of these changes were statistically significant. However, the difference between CA and CAFP at 22 h showed a reduction of 4.4% (p = 0.101) and 4.3% points (p = 0.017) per 100 μg/m^3^ for FVC and FEV_1_, respectively. None of the subjects had to use bronchodilator at any time during the study sessions or reported an increased use of bronchodilator between exposure sessions. We found no consistent changes in FEV_1_/FVC either following CA (range 70.2%-72.1%) or CAFP (range 70.9%-72.7%) exposures.

The R_AW_ at 3 h and 22 h following CA exposure showed 1.7% and −0.4% change from baseline; after CAFP the respective changes were −0.7% and 6.0%, with none of the changes being statistically significant.

**Figure 2:**
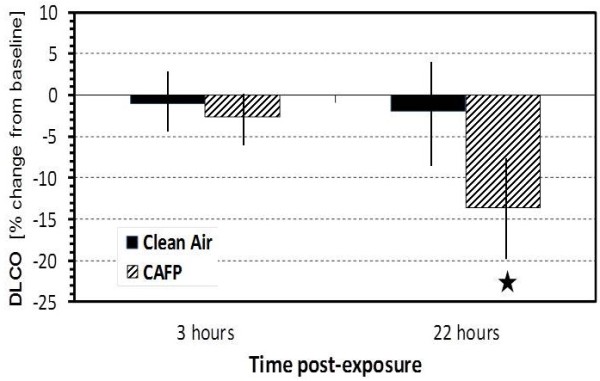
**Barplot of D**_**L**_**CO changes as % point difference from baseline measured at 3 h and 22 h post-exposure.** Black bars show estimated mean ± SEM at 0 μg/m^3^ of CA exposure and striped bars at 100 μg/m^3^ of CAFP exposure. Asterisk indicates significant difference between CA and CAFP exposures (11.1% decrease per 100 μg/m^3^, p = 0.035).

Single breath D_L_CO decreased significantly by 11.1% per 100 μg/m^3^ (p = 0.035 one sided) at 22 h following exposure to CAFP (Figure 2).

### ^99m^Tc-DTPA clearance

**Figure 3:**
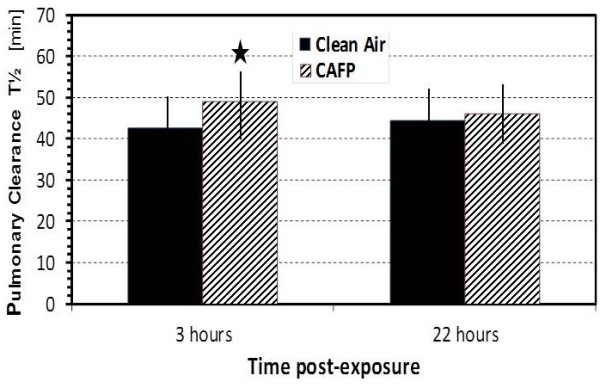
**Bar plot of pulmonary **^**99m**^**Tc-DTPA clearance half-time in measured units at 3 h and 22 h post-exposure.** Black bars show estimated mean ± SEM at 0 μg/m^3^ of CA exposure and striped bars at 100 μg/m^3^ of CAFP exposure. Asterisk indicates significant difference between CA and CAFP exposures (6.3% increase per 100 μg/m^3^, p = 0.026).

Following CA exposure mean ^99m^Tc-DTPA clearance half-time remained stable at 3 h and 22 h post-exposure at 42.6 and 44.4 min, respectively (Figure 3). Exposure to CAFP prolonged the clearance half-time at both 3 h and 22 h to estimated means of 48.9 and 46.0 min at 100 μg/m^3^, respectively. The prolongation of clearance half-time at 3 h by 6.3 min per 100 μg/m^3^ as compared to CA was statistically significant (p < 0.026). We found no significant changes in the C/P ^99m^Tc-DTPA deposition ratios at either post-CAFP exposure time.

### NO concentration

Nasal and exhaled NO concentration changes were small, inconsistent and not statistically significant at any measurement period.

### Peripheral venous blood tests

The changes in CBC with differential WBC are tabulated in Table 2. The absolute neutrophil count at 22 h post CAFP exposure was statistically significantly lower than at 3 h after CAFP exposure but not different from baseline. Neither the absolute nor percentage differential WBC count for any other type of cell was significantly different between sessions. The blood chemistry panel variables were within normal range of values at any exposure condition and sessions.

**Table 2:** Peripheral venous blood absolute cell count (mean ± SEM )

		**Pre**	**3 h post**	**22 h post**
RBC (×10^6^/μL)	Clean Air	4.30 ± 0.09	4.41 ± 0.11	4.39 ± 0.15
	CAFP	4.49 ± 0.12	4.52 ± 0.14	4.41 ± 0.13
WBC (×10^3^/μL)	Clean Air	5.29 ± 0.46	5.89 ± 0.45	5.20 ± 0.34
	CAFP	5.42 ± 0.37	6.24 ± 0.45	4.94 ± 0.23
Neutrophils (×10^3^/μL)	Clean Air	2.99 ± 0.30	3.18 ± 0.28	2.87 ± 0.20
	CAFP	3.04 ± 0.22	3.56 ± 0.35	2.67 ± 0.22*
Lymphocytes (×10^3^/μL)	Clean Air	1.74 ± 0.28	2.07 ± 0.25	1.76 ± 0.29
	CAFP	1.81 ± 0.31	2.06 ± 0.26	1.71 ± 0.28
Monocytes (×10^3^/μL)	Clean Air	0.40 ± 0.01	0.45 ± 0.03	0.38 ± 0.02
	CAFP	0.39 ± 0.03	0.45 ± 0.02	0.39 ± 0.02
Platelets (×10^3^/μL)	Clean Air	231 ± 16	241 ± 17	232 ± 13
	CAFP	258 ± 21	267 ± 23	241 ± 18

*p = 0.0326 (3 h vs. 22 h)

**Figure 4:**
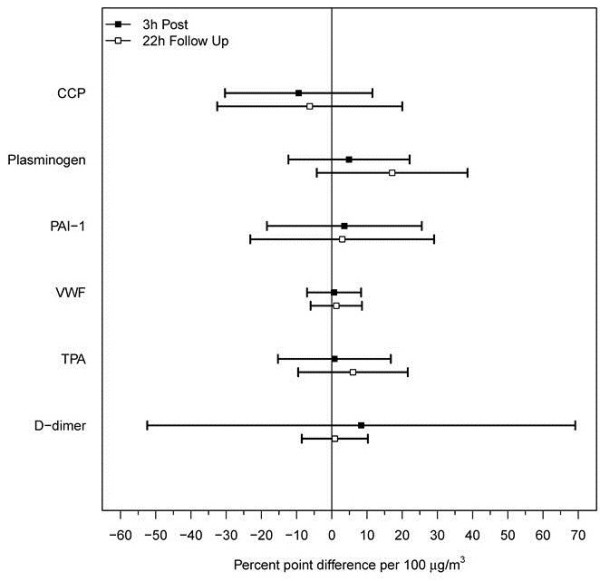
**Estimated differences between CAFP and CA exposures for selected venous blood markers of inflammation, prothrombotic activity, and lung injury measured at 3 h and 22 h post exposure.** The estimates are expressed as % point differences per 100 μg/m^3^ increase in PM concentration and relative to pre-exposure level. Horizontal bars correspond to 95% confidence intervals. None of the mediators showed a statistically significant change. For abbreviations see text.

**Figure 5:**
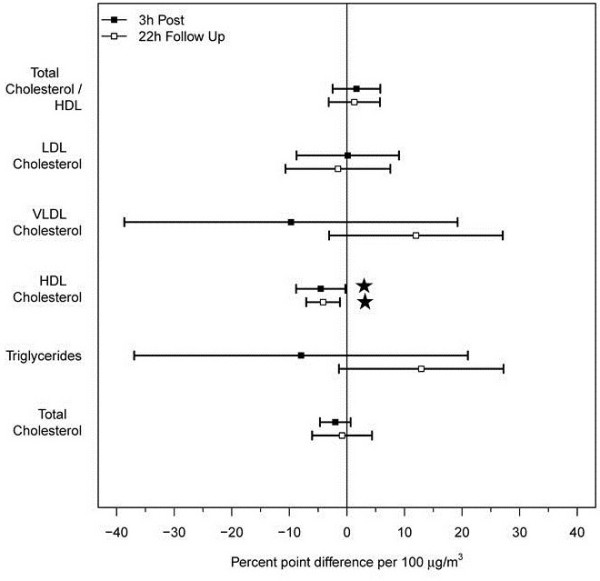
**Estimated differences between CAFP and CA exposures for selected venous blood lipids measured at 3 h and 22 h post-exposure.** The estimates are expressed as % point differences per 100 μg/m^3^ increase in PM concentration relative to pre-exposure level. Horizontal bars correspond to 95% confidence intervals. Only HDL cholesterol decreased significantly at both time points (p = 0.040 and 0.011, respectively). For abbreviations see text.

As shown in Figure 4, markers of lung injury, inflammation and procoagulant activity were not significantly affected by either the CA or CAFP exposures. However, as shown in Figure 5, HDL cholesterol was significantly reduced following CAFP relative to CA at 3 h (−4.5% per 100 μg/m^3^, p = 0.040) and 22 h (−4.1% per 100 μg/m^3^, p = 0.011) post CAFP exposure. No other lipid panel variables showed significant changes.

## Discussion

Numerous epidemiologic studies have reported various cardiopulmonary effects including increased acute morbidity and mortality due to ambient exposure of elderly individuals with COPD to fine ambient air PM [2,10,11]. Smokers at already increased risk of death from cardiopulmonary disease are even more health compromised when repeatedly exposed to PM [12]. The authors found the strongest respiratory morbidity association with ambient PM_2.5_ to have zero or one day lag. Although smoking is one of the prominent risk factors for development of COPD that can be aggravated due to exposure to air pollution [13], very few studies have explored the effects of fine PM and smoking under controlled laboratory conditions. We report a small though not statistically significant decrease in FVC and FEV_1_ in elderly smokers and subjects with mild COPD immediately after a 2 h CAFP and CA exposure at rest. Post CAFP exposure the decrements persisted and the FEV_1_ decrement became statistically significant at 22 h. The reanalysis of data after removing two ex-smokers still showed the decrease in FEV_1_ to be statistically significant the day after exposure (22 h). In a study with a similar protocol, both healthy and COPD elderly subjects exposed to CAFP during light intermittent exercise showed no statistically significant spirometric effects although the healthy individuals had a small statistically significant decrease in SpO_2_ immediately post-exposure suggesting a transient gas exchange impairment [14]. However, in a subsequent study from the same laboratory under similar conditions of exposure, healthy elderly not only showed a statistically significant decrease in SpO_2_ as in the previous study but a statistically significant decrement in FEF_25-75_ 22 h post-CAFP as well [15]. Thus, the finding of a significant decrease in FEV_1_ at 22 post-CAFP in our younger healthy smokers and ex-smokers is in general agreement with the observation of Gong et al. [15] reflecting mild impairment of large airways in young subjects and of both large and small airways in older individuals.

In healthy individuals PM_2.5_ deposits predominantly in the peripheral airways [16]. However, bronchoconstriction of conducting airways will enhance central deposition of fine particles [6]. The epidemiologic study of the Austrian Project on Health Effects of Particulate Matter (AUPHEP) with the population ranging from pre-school children to the elderly has shown that increases in R_AW_ were most consistently associated with PM_2.5_[17]. In our study we observed an insignificant increase in R_AW_ at 22 h post CAFP. Generally, the lung deposition of fine PM is higher in smokers (and even more so in COPD subjects) when compared to healthy individuals. The enhanced deposition was proportional to the severity of obstruction. In both smokers and COPD subjects the deposition fraction was negatively associated with FEV_1_ (% predicted) as well as positively associated with SR_AW_[6]. A computer simulation model showed that in COPD the overall deposition of fine PM is further increased and shifted to proximal airways as compared to healthy. Such a shift most likely increased PM dose per unit of proximal airways surface area [18]. Most likely such redistribution is due to an increased production of mucus combined with airways narrowing of smokers and individuals with COPD. We did not measure fractional lung deposition of fine PM in the present study but our pulmonary function observations are consistent with the above studies. We speculate that the decline in FEV_1_ at 22 h following CAFP exposure may be due to an enhanced deposition of fine PM in large airways, and the delay of the effect is consistent with the observation of Pope et al. [12] that the maximal respiratory effects lagged a day following exposure to fine PM.

A substantial component of PM_2.5_ is ultrafine particles (UFP) of 0.1 μm or less. These UFP are preferentially deposited in small airways. A very recent study in healthy subjects has shown that the primary UFP deposition site is the alveolar region. The deposited particles cleared very slowly with no significant elimination over several days [19]. UFP deposition (as a component of fine PM exposure) was reported to be higher in smokers when compared to healthy individuals [6]. In healthy subjects controlled exposure to 50 μg/m^3^ elemental carbon UFP resulted in a small transient increase in R_AW_, and statistically significant decrease in FEF_25-75_ and D_L_CO at 21 h post-exposure [7]. The response of our subjects to CAFP was very similar: a small increase in R_AW_, a decrease in FEV_1_ and D_L_CO at 3 h followed by even greater, statistically significant, decrease in the latter two endpoints at 22 h post exposure. Although the effects UFP may potentially have on alveolar epithelium are yet unclear it is plausible that the gas exchange function of the alveolar region may be compromised. Pietropaoli and colleagues [7] speculated that the UFP effects observed in their study of healthy individuals are due to bronchoconstriction and pulmonary vasoconstriction. Such transient effects are unlikely to have any major health consequences in healthy individuals. However, in smokers who have already reduced spirometric function and D_L_CO [20] additional reduction in spirometric function and D_L_CO may further aggravate and even limit subjects’ cardiopulmonary function and physical activity.

Although it is unclear what mechanisms may have been involved in D_L_CO reduction due to PM inhalation, a transient significant increase in DTPA clearance half-time (T_½_)(i.e., decrease in clearance rate) at 3 h post-exposure as compared to CA suggests either decreased pulmonary epithelial permeability or increased thickness of the alveolar-capillary barrier. Few studies have explored the effects of fine PM exposure on DTPA clearance. Inhalation of fine iron oxide particles (12.7 mg/m^3^) for 30 min at rest by healthy young volunteers had no significant effects on D_L_CO and DTPA clearance half-time either at 0.5 or 24 h post inhalation [21]. Prolonged 24 h chamber exposure of young healthy exercising individuals to predominantly coarse ambient PM failed to have any significant effects on the blood-gas barrier as measured by DTPA clearance rate [22]. Other studies, however, have shown increased epithelial permeability as assessed by DTPA clearance rate in smokers vs. nonsmokers [23,24]. Interpretation of these and of our findings is complicated by likely differences in pre-existing chronic inflammation and changes in airway function and morphology associated with chronic smoking. Evidence for chronic inflammation in our subjects is supported by baseline their DTPA clearance half-time being more than twice as rapid as what we observed previously in healthy non-smoking subjects [21] (mean halftime of 43 vs. 105 min in non-smokers). We speculate that, in our study, fine PM depositing into likely inflamed airways and UFP depositing primarily in the alveolar region induced a transient interstitial edema, effectively thickening the blood-gas barrier which resulted in reduced D_L_CO and an increased DTPA clearance half-time. This interpretation is supported by the work of Foster et al. [25] which showed that mild airway wall edema due to inflamed airways in sheep reduced the rate of DTPA clearance rate.

The significant respiratory response contrasts with minimal effects on a large number of measured peripheral blood endpoints (coagulation factors, inflammatory mediators, and blood cell count, blood chemistries and lipids). With the exception of changes in HDL we did not find any meaningful associations between the changes in blood variables, PM concentration or pulmonary function changes. Considering a relatively short exposure time at rest it is unlikely that the concentration of the particles was high enough to induce systemic effects. A similar study with a higher level of CAFP and inclusion of light exercise also reported a lack of significant changes in CBC, differential WBC and procoagulation factors [14]. On the other hand, Ghio et al. [8] reported neutrophilia in younger individuals exposed to Chapel Hill CAFP for 2 h while alternating 15 min exercise (minute ventilation of about 50 L/min) with 15 min rest to a slightly higher PM_2.5_ concentration (120.5 ± 14 μg/m^3^) than in our study. In our middle-aged cohort we observed only a small increase in absolute WBC at 3 h post exposure to both CA and CAFP. The absence of consistent inflammatory, coagulation and blood cell count changes suggest that the respective mechanisms were not sufficiently activated by relatively low fine PM exposure load in our study. Even in studies of elderly where the average fine PM concentration was twice as high as ours the induced changes in the above endpoints were minimal if any [14,15]. In our cohort of 11 smokers we did not observe any enhanced pulmonary or inflammatory response in those individuals either. Very recently, however, Rice et al. [26] reported a significant decrease in circulating HDL cholesterol level but no other lipids in welders acutely exposed to primarily PM_2.5_ welding fumes. Our results parallel these findings, also showing a significant post-exposure decrease in HDL at 3 and 22 h without any significant changes in other lipids.

## Conclusions

We found that in 11 middle-aged to elderly mostly female smokers, including two ex-smokers, there were statistically significant lung function decrements after a 2 h exposure to concentrated fine Chapel Hill PM (CAFP) at rest. With respect to our a priori primary endpoint measures we found a significant reduction in FEV_1_ at 22 h post-exposure and a significant increase in DTPA clearance half-time at 3 h post-exposure. The coherence of these observations contrasts with randomness of response in various exploratory endpoints – serum chemistries and protein panels, and procoagulants. These changes were not driven by two ex-smokers since the removal of their data did not change the statistical significance of these primary outcomes. These findings are consistent with mild airways inflammation and plausibly with transient interstitial edema. The findings in other exploratory endpoints such as hematologic, serum chemistry and protein panels, and procoagulants appeared to be random. The lack of consistent inflammatory response makes it difficult to assess the systemic significance of the observed effects. Since these effects developed after resting inhalation of relatively low concentrations of CAFP the lung function changes might be expected to be more pronounced in individuals with pre-existing conditions, with higher inhaled CAFP concentrations and with increased ventilation attendant to exercise.

## Methods

### Subjects

Seventeen current and ex-smokers, aged 35–74 yrs, recruited from the general population who initially qualified for the study approved by the UNC IRB and the EPA but for various technical reasons only 11 participated in the exposure phase of the study. On the day of exposure and until the completion of the study and discharge the next day subjects were not allowed to smoke but were allowed to use their own nicotine gum/patch.

### Protocol

Eligible volunteers came to the laboratory 5 times; a training day and two exposure days with 22 h follow-up separated by at least 3 weeks. Double-blind and counterbalanced exposures to CA or CAFP with an MMAD of less than 2.5 microns lasted 2 h with subjects resting during the exposure. During the training session the subjects were familiarized with and performed most of the study procedures. As part of this session ECG leads for 24 h Holter monitoring were placed on the subjects (Mortara, Milwaukee, WI). Subjects were excluded from further study if the Holter reviewed by a cardiologist showed significant arrhythmia or evidence of ischemia.

Upon arrival in the laboratory on the exposure day, medical personnel ascertained the subject’s general health, took vital signs and evaluated respiratory symptoms. Subsequently, the subjects performed pre-exposure lung function tests, had ECG electrodes attached for safety monitoring, had blood samples drawn and entered the exposure chamber with conditions blinded to both the subject and the investigator. ECG and finger pulse S_p_O_2_ were monitored continuously during the exposure and spirometric measures of lung function were checked midway through the exposures. Subjects were asked to refrain but were not prohibited from using inhaled bronchodilators during exposures; if medication use was necessary subjects were instructed to maintain consistent medication use across the two exposures. After completing post-exposure testing and blood draw the subjects were discharged. The next morning subjects returned to the facility for 22 h post exposure testing.

### Exposure chamber and PM generation

All exposures were carried out at the EPA Human Studies Facility (HSF) on the University of North Carolina campus at Chapel Hill, NC. A fine particle concentrator [27] installed at the EPA HSF and described earlier [28] was used for this study.

### Procedures

Spirometry was measured before, immediately, 3 h and 22 h after exposure using Sensormedics Vmax 229 system (Yorba Linda, CA) conforming to recommended ATS/ERS procedure [29].

The permeability of the respiratory epithelium was assessed at 3 h and 22 h post exposure by monitoring the pulmonary clearance of an inhaled radiolabeled tracer molecule (^99m^Tc-DTPA) from the lungs into the blood using a gamma camera. The rate of clearance of radioactivity from the thorax serves as an index of alveolar epithelial permeability and the results are reported as the calculated clearance half-time (T_1/2_) [21]. Regional deposition within the central (C) and peripheral (P) regions of the lung was examined by calculating a central to peripheral (C/P) ratio based on counts from the initial one minute dynamic image following deposition of the inhaled radiolabeled DTPA aerosol [30].

As secondary endpoints, we measured D_L_CO [31] and plethysmographic airway resistance using Sensormedics Vmax system, and exhaled (bag collection) and nasal (on-line) nitric oxide [32] using Sievers 270B analyzer (GE Analytical, Boulder, CO).

Blood was drawn immediately prior to and both 3 h and 22 h after exposure to obtain peripheral venous blood cell counts and blood chemistry measurements including a complete blood count (CBC), circulating levels of inflammatory cytokines, serum electrolytes, lipoproteins (triglycerids, total cholesterol, HDL, LDL) and indicators of kidney (creatinine, BUN, BUN/creatinine ratio) and liver (bilirubin, total protein, albumin, globulin, ALP, AST-SGOT, ALT-SGPT, GGT) function. Plasma assays included tPA, PAI-1, vWF, CRP, quantitative CRP, Clara cell 16 protein (CCP), and D-dimer.

### Statistical approach

The primary variables of interest selected a priori were FEV_1_ and ^99m^Tc-DTPA clearance. The other reported endpoints were of secondary interest. All endpoints with the exception of ^99m^Tc-DTPA clearance were acquired at baseline (pre-exposure), 3 h and 22 h post-exposure. Spirometry was also measured immediately post-exposure while DTPA clearance was only measured at 3 h and 22 h post-exposure. The endpoints with baseline values were expressed as % of baseline (100*post/pre) to control for day-to-day variability. The differences in responses between CA and CAFP were examined using linear mixed effects models with subject-specific random intercepts to account for repeated measures and subject CAFP exposure level variability. The differences in responses between CA and CAFP were considered separately for each time point (immediately post, 3 h and 22 h post-exposure). They were expressed per 100 μg/m^3^ of PM relative to baseline in normalized endpoints (spirometry, D_L_CO and blood data) and in measured units for ^99m^Tc-DTPA clearance half-time. R statistical software (Version 2.11.1) was used for the analysis and to generate plots. To determine statistical significance at α = 0.05 two-tailed test was used to evaluate all data except D_L_CO where one-tailed test was used.

In this study a large number of endpoints were measured and so a question of multiple comparisons correction is of concern. For the primary endpoints (FEV_1_, DTPA) multiple comparison adjustment is not appropriate since they were declared as such a priori and are not covariates. While the study was powered on FEV_1_ changes observed in other studies we measured a number of secondary endpoints not studied under these conditions in this population. Among approximately 40 blood exploratory endpoints, only one (HDL) was found to have a p-value <0.05. The other one was D_L_CO. The seemingly significant change of these two endpoints might be due to chance rather than due to CAFP exposure. After adjusting for multiple testing, the HDL and D_L_CO changes would not have been statistically significant. However, the small sample size may have an impact on the rate of false negative findings particularly when CAFP-induced changes may be small.

## Abbreviations

ALP: Alkaline phosphatase; AST-SGOT: Aspartate aminotransferase; ALT-SGPT: Alanine aminotransferase; BUN: Blood urea nitrogen; CA: Clean air; CAFP: Concentrated ambient fine particulates; CBC: Complete blood count; CCP: Clara cell 16 protein; COPD: Chronic obstructive pulmonary disease; CRP: C-reactive protein; DLCO: Lung diffusing capacity for carbon monoxide; ECG: Electrocardiogram; FEF25-75: Forced expiratory flow between 25-75% of FVC; FEV1: Forced expiratory volume in 1 sec; FVC: Forced vital capacity; GGT: Gamma glutamyl transferase; HDL: High density lipoprotein; LDL: Low density lipoprotein; PAI-1: Plasminogen activator inhibitor; PM: Particulate matter; SpO2: Pulse oximeter oxygen saturation; RAW: Airway resistance; SRAW: Specific airway resistance; 99mTc-DTPA: Technetium-99m diethylene triamine pentaacetic acid; tPA: Tissue plasminogen activator; vWF: Von Willebrand factor antigen level; UFPM: Ultrafine particulate matter.

## Competing interests

The authors declare that they have no competing interests.

## Authors’ contributions

Conception and design: MJH, PAB, RBD, WB, NEA, WEC. Acquisition of data: MJH, WB, JCL, KZ, HK. Analysis and interpretation of data: MJH, AGR, NA, PAB, JL, RBD Drafting and revisions of the manuscript: MJH, PAB, JL, WB, NA, AGR, WEC, RBD. All authors read and approved the final manuscript.
